# Single-cell RNA sequencing reveals sexual diversity in the human bladder and its prospective impacts on bladder cancer and urinary tract infection

**DOI:** 10.1186/s12920-023-01535-6

**Published:** 2023-06-05

**Authors:** Ribao Wu, Xiahong Teng, Qiong Song, Shuai Chen, Lihui Wang, Jinling Liao, Chunlin Zou

**Affiliations:** 1 Center for Translational Medicine, Key Laboratory of Longevity and Aging-related Diseases of Chinese Ministry of Education, Institute of Neuroscience and Guangxi Key Laboratory of Brain Science, School of Basic Medical Sciences, Guangxi Medical University, Nanning, Guangxi China; 2 School of International Education, Guangxi Medical University, Nanning, Guangxi China; 3 Center for Genomic and Personalized Medicine, Guangxi Medical University, Nanning, Guangxi China

**Keywords:** ScRNA-seq, Sex-based differential gene, Urinary tract infection, Bladder cancer, Extracellular matrix

## Abstract

**Background:**

Some bladder-related diseases, such as bladder urinary tract infection (UTI) and bladder cancer (BCa), have significant six differences in incidence and prognosis. However, the molecular mechanisms underlying these sex differences are still not fully understood. Understanding the sex-biased differences in gene expression in normal bladder cells can help resolve these problems.

**Methods:**

We first collected published single-cell RNA sequencing (scRNA-seq) data of normal human bladders from females and males to map the bladder transcriptomic landscape. Then, Gene Ontology (GO) analysis and gene set enrichment analysis (GSEA) were used to determine the significant pathways that changed in the specific cell populations. The Monocle2 package was performed to reconstruct the differentiation trajectories of fibroblasts. In addition, the scMetabolism package was used to analyze the metabolic activity at the single-cell level, and the SCENIC package was used to analyze the regulatory network.

**Results:**

In total, 27,437 cells passed stringent quality control, and eight main cell types in human bladder were identified according to classical markers. Sex-based differential gene expression profiles were mainly observed in human bladder urothelial cells, fibroblasts, B cells, and T cells. We found that urothelial cells in males demonstrated a higher growth rate. Moreover, female fibroblasts produced more extracellular matrix, including seven collagen genes that may mediate BCa progression. Furthermore, the results showed that B cells in female bladders exhibited more B-cell activated signals and a higher expression of immunoglobulin genes. We also found that T cells in female bladders exhibited more T-cell activated signals. These different biological functions and properties of these cell populations may correlate with sex differences in UTI and BCa, and result in different disease processes and outcomes.

**Conclusions:**

Our study provides reasonable insights for further studies of sex-based physiological and pathological disparities in the human bladder, which will contribute to the understanding of epidemiological differences in UTI and BCa.

**Supplementary Information:**

The online version contains supplementary material available at 10.1186/s12920-023-01535-6.

## Background

The urinary bladder is an integral part of the human urinary system. When bladder cells are dysfunctional, there are increased chances of developing bladder-related disorders [1]. Bladder-related disorders generate substantial economic burdens each year. For example, urinary tract infection (UTI) is one of the most prevalent bacterial illnesses, affecting 150 million individuals worldwide each year and creating a medical burden of more than $6 billion [2]. In addition, bladder cancer (BCa) is the most common malignancy of the urinary tract, with a high incidence and expensive treatment cost [3].

Notably, there are considerable gender differences in UTIs and BCa. For example, females are more susceptible to UTIs than males, and adult premenopausal women are 40 times more likely to develop UTIs than adult men [4]. Regarding BCa, the number of male patients affected by BCa is usually three to four times greater than that of female patients [5]. Moreover, women diagnosed with BCa are more likely to have locally advanced tumors at the time of diagnosis [6].

However, the molecular mechanisms of the sex diversities in bladder-related disorders, even in the normal bladder, are still not fully understood. Single-cell RNA sequencing (scRNA-seq) is a highly quantitative and sensitive technology that provides the comprehensive landscape of cellular components in different organs and new avenues to address these questions. To uncover sexual diversity in the normal human bladder, we collected previous scRNA-seq data [1, 7, 8] to map the human bladder transcriptomic landscape in both females and males. Sex-based differential gene expression profiles were mainly observed in urothelial cells, fibroblasts, B cells, and T cells in the human bladder. Our results showed that the pathways associated with ribosome biogenesis were enriched in urothelial cells in the male bladders, and fibroblasts in the female bladders expressed more extracellular matrix (ECM), including seven collagen genes (*COL1A1*, *COL1A2*, *COL3A1*, *COL5A2*, *COL6A1*, *COL6A2*, and *COL6A3*) associated with a poor prognosis of BCa. Moreover, we found that B cells and T cells in the female bladders exhibited more activated signals. These sexual diversities confer different biological properties to the female and male bladder, which may correlate with sex differences in UTI and BCa and result in different disease processes and outcomes.

In conclusion, our study provides the cellular landscape and sex-based gene expression differences in the normal human bladder at a single-cell resolution. Our work provides information enhancing our understanding of sex-based physiological and pathological disparities in the normal human bladder and some bladder-related diseases.

## Materials and methods

### Analysis of BCa transcriptome sequencing data from the TCGA database

The transcriptome sequencing data and clinical data of BCa were downloaded from TCGA database using the TCGAbiolinks package (version 2.22.4). Then, we used the “wilcox.test” method to statistically analyze the expression of some collagen genes in different stages. Data from stage I were excluded because of the limited number of samples (only four samples). Finally, the results were visualized by the ggplot2 package (version 3.3.6).

### scRNA-seq data collection and data analysis

The scRNA-seq data of six normal human bladders were collected from the public databases (Supplementary Table 1). Cell Ranger (version 6.1.2) was used for the raw fastq data analysis and to generate the gene count matrices with the default parameters. After that, we used the R package (version 4.1.2) and Seurat package (version 4.1.0) to load the gene count matrices and created a Seurat object for each one. Then, we used different criteria, such as gene counts of a cell (nFeature_RNA), percentage of mitochondrial genes (mt.pct), and percentage of hemoglobin genes (hb.pct), to remove low-quality or dead cells (Supplementary Table 2) depending on the specifics of each sample (Supplementary Fig. 1A-C). All Seurat objects were then combined using the “merge” function. After data normalization and cell cycle scoring, we used the “FindVariableFeatures” function with the default parameters to calculate the highly variable genes. Next, a principal component analysis (PCA) was performed with the “RunPCA” function based on these variable genes. Subsequently, we removed the batch effect of sequencing via the “RunHarmony” function in the harmony package (version 0.1.0). In total, 15 principal components were selected for T-distributed Stochastic Neighbor Embedding (TSNE). Then, we used the “FindClusters” function to identify cell clusters. Finally, we used the “FindAllMarkers” function to find differentially expressed genes (DEGs) among each cluster of cells.

### Differentially expressed genes (DEGs) analysis and functional annotation

First, we extracted the different combinations of cell populations by the “subset” function for further sex-biased analysis. Then, we used the “FindMarkers” function to identify DEGs (female vs. male). Log2FC is the fold change of gene expression in log2 scale, and DEGs were identified by “|Log2FC| > 0.5 with p.adjust < 0.05” in this study. The Gene Ontology (GO) analysis and gene set enrichment analysis (GSEA) were performed using the clusterProfiler package (version 4.2.2) to determine the significant pathways. Especially, GSEA analysis was perform with genes ranked by log difference in average expression between females and males.

### SCENIC (single-cell regulatory network inference and clustering) analysis

SCENIC is a method of reconstructing gene regulatory networks and identifying cell states based on co-expression and DNA motifs [9]. In our study, the SCENIC package (version 1.2.4) was used to infer gene regulatory networks and the key transcription factors (TFs) of urothelial cells and fibroblasts. We first eliminated genes expressed only in a few cells (< 1%) and obtained genes available on the RcisTarget database. Next, we used the “runCorrelation” function to obtain the correlation matrix of genes and identified the potential targets of each transcription factor by running GENIE3. Then, potential direct targets were identified by running RcisTarget. Finally, we identified the cellular states of individual cells by analyzing and scoring the intracellular network activity using AUCell. Subsequently, we imported the results of the SCENIC analysis into the Seurat object for further transcriptional regulatory network analysis and utilized the “FindMarkers” function to identify the differential TFs in various cell populations.

### scMetabolism analysis

scMetabolism is an R package that scores the metabolic activity of each cell based on VISION, AUCell, ssgsea, and gsva algorithms, and finally obtains the activity score of cells in each metabolic pathway [10]. In our study, the urothelial cells were extracted from the Seurat object to compare sex-based metabolic disparities. We used the VISION quantification method in this package to evaluate the metabolic activity of each cell and finally obtained the activity score of the cells in each metabolic pathway (KEGG pathway). To further determine whether sex-based disparities exist in these metabolic pathways, the bimod3 test was used for the differential analysis.

### Single cell trajectory analysis

Monocle is an algorithm that uses reverse graph embedding to describe multiple destiny decisions in a completely unsupervised manner [11]. To construct a single cell pseudotime trajectory and cell fate decisions and identify genes that change as cells undergo transition, the monocle2 package (version 2.20.0) was applied to the cells from fibroblasts in this study. These fibroblasts were separated into male and female subgroups, and pseudotime trajectory reconstruction was performed independently using the monocle2 package. First, the size factors and dispersions were obtained from the “estimateSizeFactors” and “estimateDispersions” functions. Then, we adopted a method recommended by monocle to identify order genes with a high degree of dispersion (mean_expression > = 0.01 and dispersion_empirical > = dispersion_fit). These order genes were used to reduce dimension and order cells. Subsequently, dynamic genes along the pseudotime and states were plotted on maps by the ‘plot_genes_in_pseudotime’ function.

### Cell type specific expression of BCa-associated genes

To compare gender disparities in the expression of BCa-associated genes in specific bladder cell types, the “AverageExpression” function was applied to obtain the average expression matrices of BCa-associated genes in female and male bladder cells. Then, we used the pheatmap package (version 1.0.12) to visualize these matrices.

## Results

### scRNA-seq profiling of normal adult human bladder

**Fig. 1:**
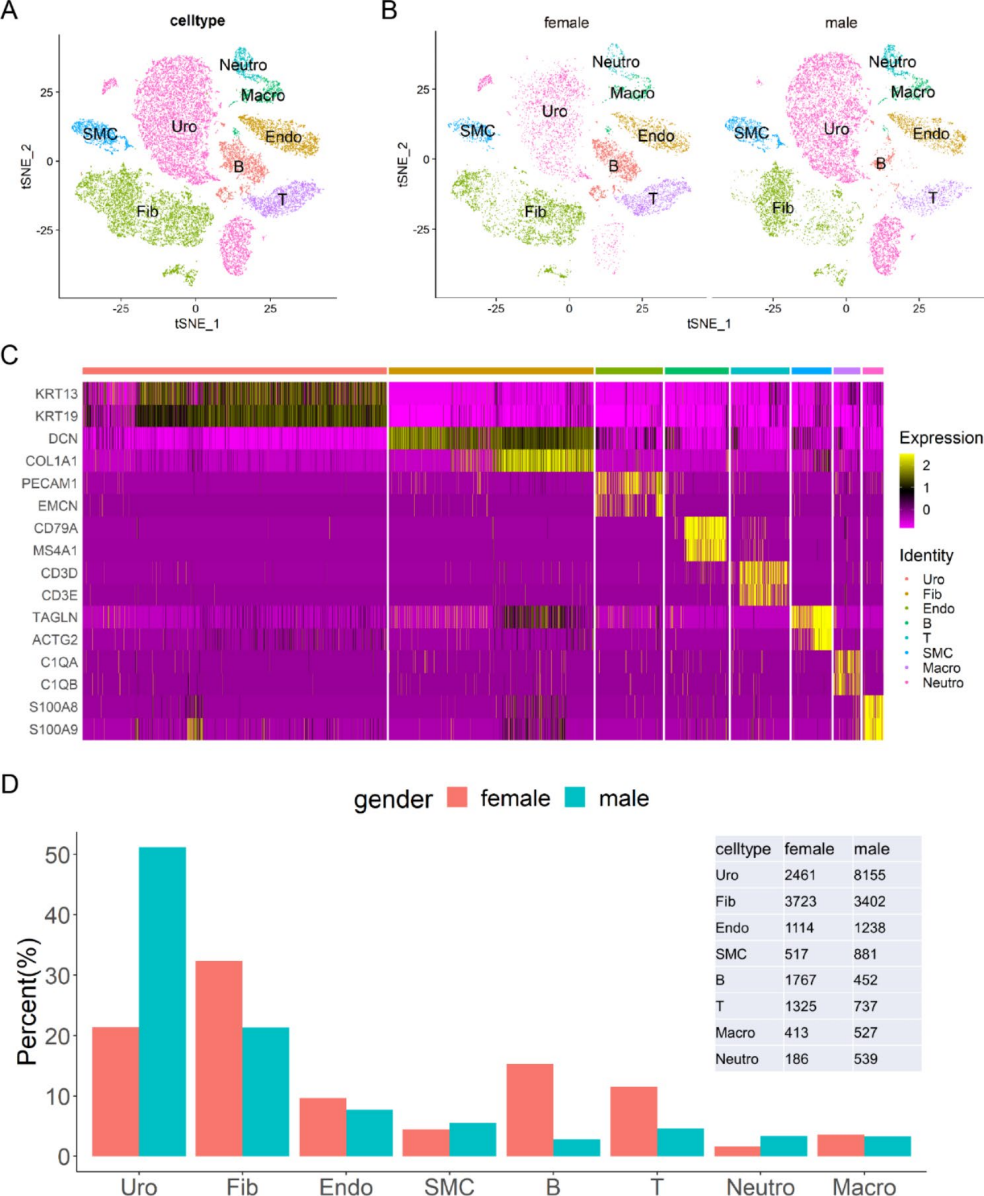
scRNA-seq landscape of human bladder cells. (**A**) TSNE map showing eight distinct cell types in the human bladder. Uro: urothelial cell, Fib: fibroblast, Endo: endothelial cell, SMC: smooth muscle cell, Macro: macrophage, Neutro: neutrophil, B: B cell, T: T cell. (**B**) TSNE map showing sex-based cell distribution differences in the human bladder. (**C**) Heatmap showing the marker genes of each cell type. (**D**) Bar plot showing the proportions of different cell types in female bladders and male bladders. The cell number of each cell type is provided in the right panel

Cells from six human bladders, including three males and three females, were collected from published data. In total, 44,728 cells were obtained from all samples. After stringent quality control by Seurat, 27,437 high-quality cells were retained for further analysis. According to cell-specific marker genes (Table 1), we identified eight cell types, corresponding to urothelial cells (Uro), fibroblasts (Fib), endothelial cells (Endo), smooth muscle cells (SMC), macrophages (Macro), neutrophils (Neutro), B cells, and T cells (Fig. 1A and C). Similarities and dissimilarities between the male and female bladder cells were observed in the TSNE analyses (Fig. 1B). We further analyzed the proportion of different cell types between female and male bladder cells. The results showed that sex differences in cell proportions mainly existed in urothelial cells, fibroblasts, B cells, and T cells (Fig. 1D). These proportion differences based on sex suggest that these four cell types may be associated with sexual diversity in the normal human bladder.

**Table 1:** Classical marker genes in different cell types

Cell type	Marker genes
Uro	KRT13, KRT19
Fib	DCN, COL1A1
Endo	PECAM1, EMCN
SMC	TAGLN, ACTG2
B	CD79A, MS4A1
T	CD3D, CD3E
Macro	C1QA, C1QB
Neutro	S100A8, S100A9

### Sex-related diversity in urothelial cells at a single-cell resolution

**Fig. 2:**
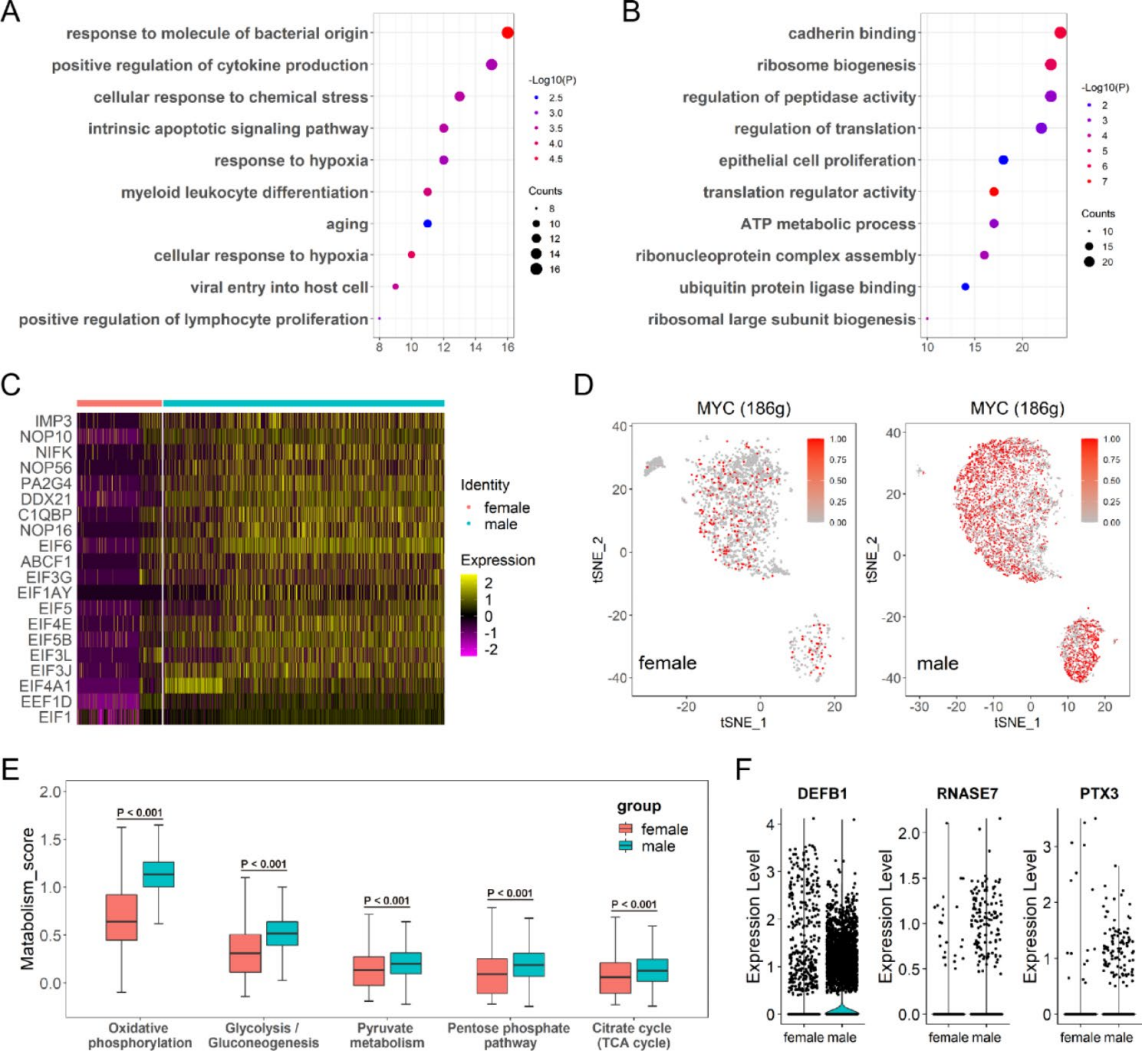
Sex-related diversity of urothelial cells in the human bladder. (**A**) Bubble plot showing ten representative GO pathways in female urothelial cells. (**B**) Bubble plot showing ten representative GO pathways in male urothelial cells. (**C**) DEG analysis showing ribosome biogenesis-related genes upregulated in urothelial cells in male bladders. (**D**) SCENIC analysis showing the activity of the transcription factor *MYC* in urothelial cells of females and males. The depth of the red represents the degree of activity. (**E**) Boxplot showing enrichment scores of representative metabolic pathways in male and female urothelial cells. (**F**) Violin plots demonstrating three antimicrobial compounds are predominantly expressed in urothelial cells in males

To explored the sex-based heterogeneity of urothelial cells in the human bladders, we first performed GO analysis base on the DEGs of urothelial cells. The results of the GO analysis showed that the genes upregulated in female urothelial cells were mainly associated with the response to stimuli by bacteria and intrinsic apoptotic signals (Fig. 2A). In contrast, genes and pathways related to ribosome biogenesis were significantly upregulated in males (Fig. 2B and C). Ribosome biogenesis is a fundamental process closely coordinated with cell growth and proliferation [12]. Therefore, we hypothesized that male urothelial cells might be a population of actively growing cells compared to female urothelial cells. In order to compare the growth activity of urothelial cells between females and males, a cell cycle analysis was performed on individual cells. The results showed that 55.1% of the male urothelial cells were in the S or G2/M phase, compared with 35.2% in female urothelial cells (Supplementary Fig. 2A and B). We also found that the percentage of *PCNA*-positive cells in male urothelial cells was higher than that in female urothelial cells (Supplementary Fig. 2 C and D), suggesting that more proliferating cells exist in urothelial cells in males. Furthermore, the SCENIC analysis revealed that the transcription factor *MYC* was more active in male urothelial cells (Fig. 2D). *MYC* is a major transcription factor involved in regulating cell proliferation and metabolism, and promoting cell growth and malignant transformation [13, 14]. The results of the scMetabolism analysis also showed that male urothelial cells were more active in energy metabolism than female urothelial cells, especially in glucose utilization, which is consistent with the high energy consumption of ribosome biogenesis (Fig. 2E). In addition, we compared several important antimicrobial compounds (*DEFB1*, *RNASE7*, and *PTX3*) that appear more prevalent in men (Fig. 2F).

### Sex-related diversity in fibroblasts at a single-cell resolution

**Fig. 3:**
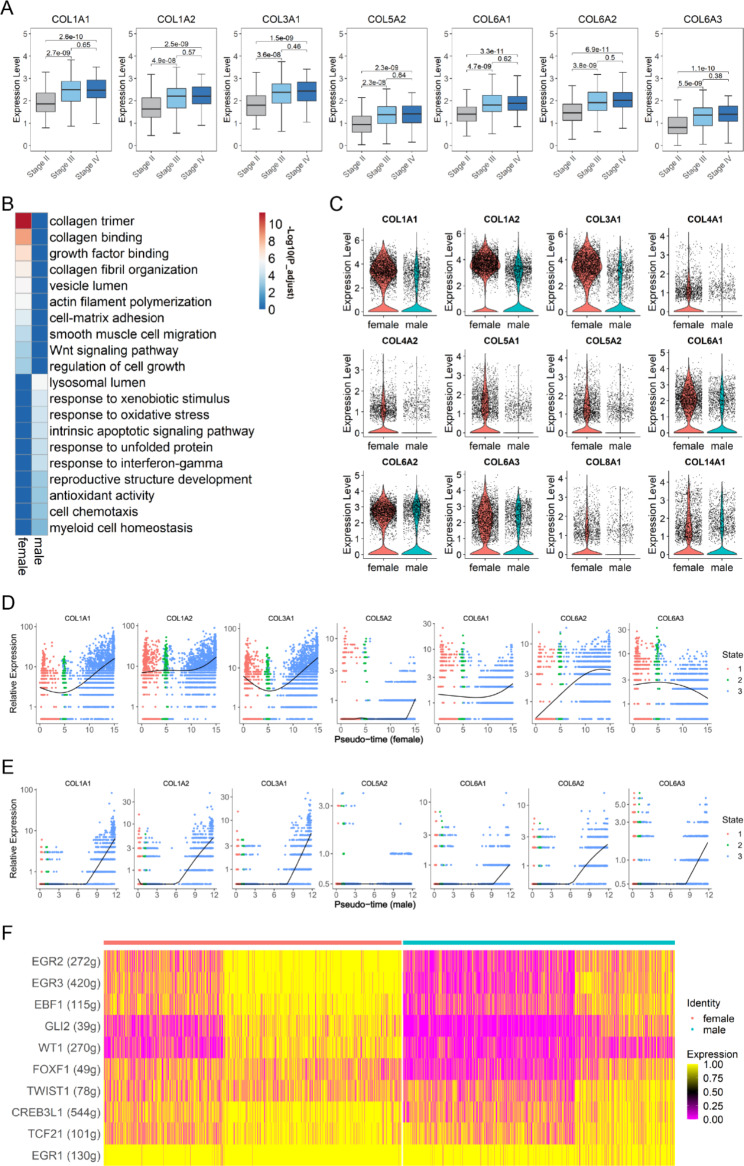
Sex-related diversity of fibroblasts in the human bladder. (**A**) Boxplots showing that seven collagen genes are upregulated in the advanced stages of BCa. (**B**) Heatmap demonstrating representative GO pathways of the fibroblasts of females and males based on a DEG analysis between females and males. (**C**) Violin plots showing that 12 ECM genes are predominantly expressed in female fibroblasts. (**D-E**) Pseudotime trajectory analysis of seven BCa-related ECM genes in fibroblasts from females (**D**) and males (**E**). The results show that the relative expression levels of these seven collagen genes were higher in female fibroblasts than in male fibroblasts during the process of fibroblast differentiation. (**F**) SCENIC analysis showing that most transcription factors of seven BCa-related ECM genes exhibit higher activity in female fibroblasts

In collagen-dense tumors, tumor cells can induce collagen fiber remodeling in paracancerous tissues, such that collagen fibers are arranged radially along with tumor tissues, allowing tumor cells to migrate along these radially aligned fibers and invade the surrounding tissues [15]. For example, collagens, such as *COL1A1*, *COL1A2*, *COL3A1*, *COL5A2*, *COL6A1*, *COL6A2*, and *COL6A3* are thought to mediate tumor progression and are associated with a poor prognosis in BCa [16, 17]. In our study, by analyzing transcriptome sequencing data of BCa from TCGA database, we found that these seven collagen genes were upregulated in advanced stages, suggesting that they might promote the progression of BCa (Fig. 3A).

In addition, the results of GO analysis showed that pathways associated with the ECM were significantly upregulated in female bladder fibroblasts (Fig. 3B). Twelve collagen genes were expressed at higher levels in female fibroblasts than in males, including seven genes associated with a poor prognosis of BCa, suggesting that female fibroblasts may play a more significant role in promoting metastasis and progression than male fibroblasts (Fig. 3 C). We further explored the reasons for this differential expression. The results of the trajectory analysis showed that the relative expression levels of these seven collagen genes in the pseudotime trajectory of female fibroblasts were significantly higher than that in males (Fig. 3D and E, Supplementary Fig. 2E and F). Three genes (*COL5A2*, *COL6A1*, and *COL6A3*) were barely expressed in the pseudotime trajectory of male fibroblasts or only slightly upregulated at the end of pseudotime trajectory. We also performed a SCENIC analysis, and the results showed that most TFs associated with the expression of these collagen genes were more active in female fibroblasts than in males (Table 2; Fig. 3F).

**Table 2:** Transcription regulators for ECM genes

TF	Target genes
EGR1	COL1A1, COL1A2, COL3A1, COL6A1
EGR2	COL1A1, COL6A1, COL6A3
EGR3	COL1A1, COL6A1, COL6A3
CREB3L1	COL1A1, COL1A2, COL3A1
WT1	COL1A1, COL5A2, COL6A1
TWIST1	COL1A1, COL6A1, COL6A3
EBF1	COL1A1, COL5A2
GLI2	COL3A1
TCF21	COL3A1
FOXF1	COL6A3

### Females exhibited a higher expression of B-cell and T-cell activated signaling at the transcript level

**Fig. 4:**
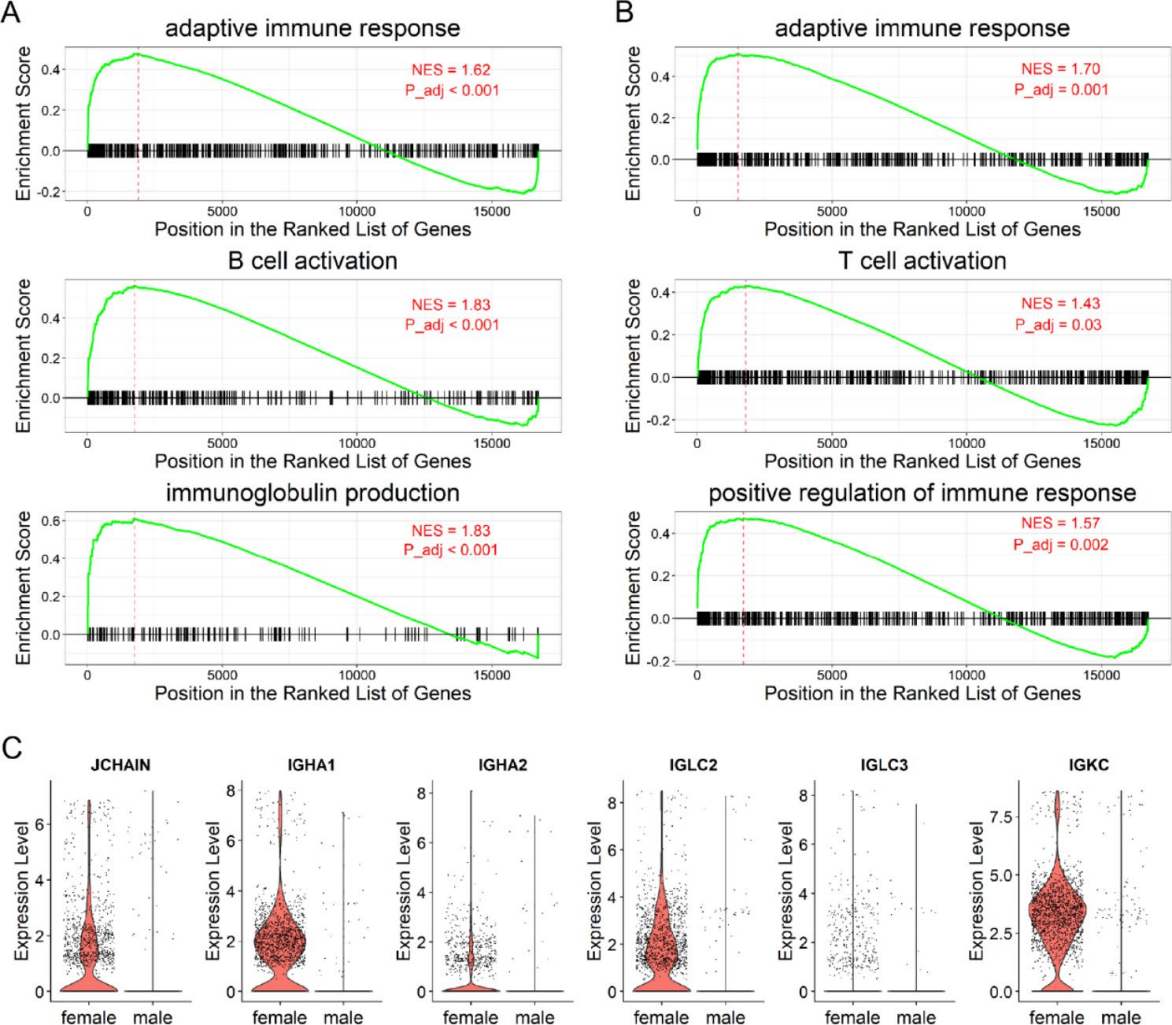
Females exhibit a higher expression of B-cell and T-cell activated signals. (**A-B**) GSEA showing that females exhibit higher expression of B-cell activated signals (**A**) and higher expression of T-cell activated signals (**B**) than that in males. The normalized enrichment scores (NES) and adjust P value (P_adj) were showed on enrichment plots. When NES > 0 and P_adj < 0.05, it indicates that this pathway is enriched in females. (**C**) Violin plots demonstrating that six immunoglobulin genes are predominantly expressed in the B cells of females

Sex affects the human immune system, leading to different responses to infection, autoimmunity, and cancer [18]. Our results showed that more B cells and T cells are present in the female bladder, suggesting that different adaptive immune responses may exist in the bladder between females and males. The GSEA results showed that females exhibited higher B-cell and T-cells activated signals at the transcriptional level (Fig. 4A and B). We found that the expression of immunoglobulin genes (such as *JCHAIN*, *IGKC*, *IGLC3*, *IGHA1*, *IGLC2*, and *IGHA2*) in female B cells was higher than that in males B cells (Fig. 4 C).

Notably, although the incidence of female UTIs in adulthood is significantly higher than that in men, male UTI patients have an elevated risk of UTI-associated complications, as infection in men of all ages is clinically classified as a complicated UTI [19]. In a mouse model of UTI, female bladders could quickly initiate an immune response to clear the bacteria promptly when UTIs occurred. In contrast, the male immune response was unable to clear the bacteria completely and displayed persistent bacteriuria [4], which is similar to UTIs in humans. For example, although female and male patients are given the same first-line antibiotics, men require more prolonged treatment to eradicate the bacteria [20]. Our study may partially explain this phenomenon. Compared to male bladders, female bladders may exist more plasma cells (marked by *JCHAIN* and *IGKC*), allowing them to produce more immunoglobulins. Once UTI occurs, these immunoglobulins may be able to recognize the pathogen more quickly and activate other immune cells to clear the bacteria in time to maintain homeostasis in the body.

### Sex-related diversity in the expression patterns of bladder cancer-related genes

**Fig. 5:**
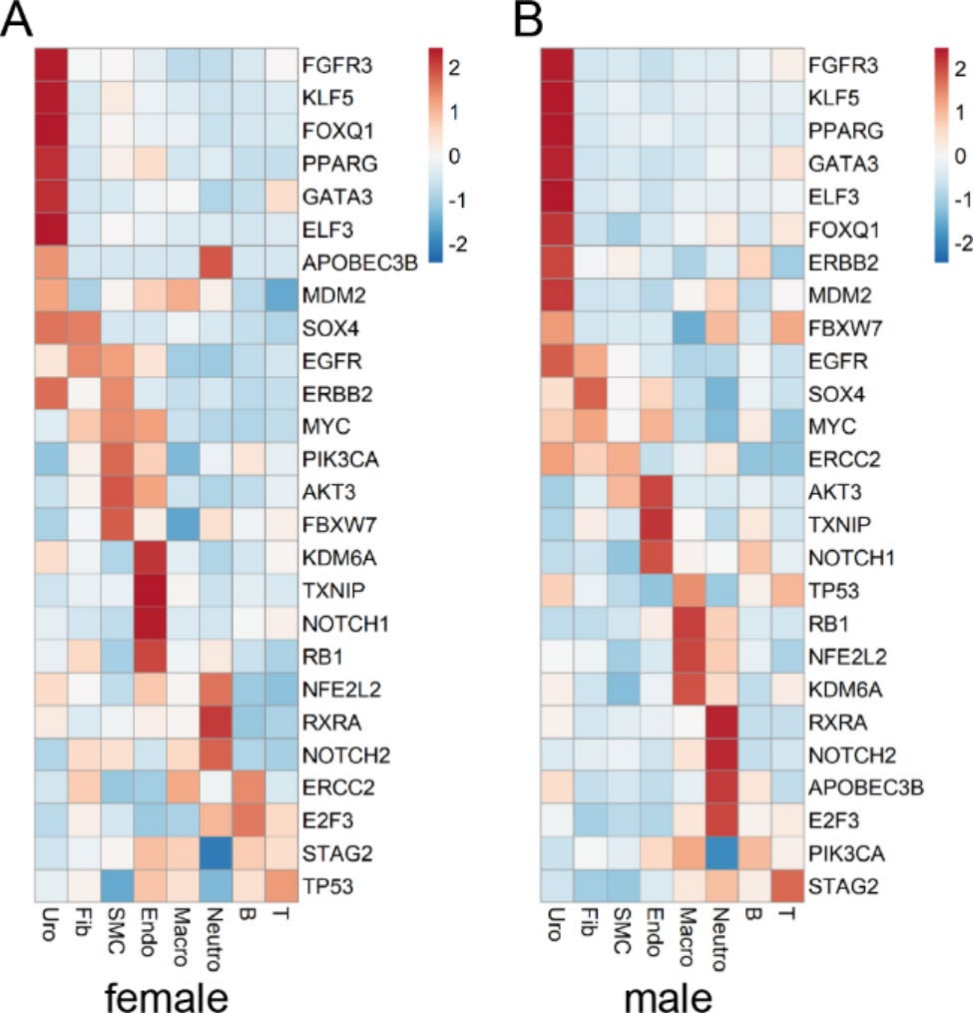
Six diversity of BCa-associated genes in human bladders. (**A-B**) Heatmap showing the expression of BCa-associated genes in female bladder cells (**A**) and male bladder cells (**B**). The mean expression values of these genes were calculated by the “AverageExpression” function in each cell type

BCa includes the following three main pathological types: urothelial bladder carcinoma (UBC), squamous cell carcinoma, and adenocarcinoma [21]. According to several reports, BCa originates from urothelial cells, and UBC accounts for approximately 90% of BCa [5, 22]. Although BCa is a malignancy with significant gender differences, there appears to be no significant gender difference in the type of BCa pathology. However, women are diagnosed with more advanced disease at presentation and have poor prognoses. This gender commonality and disparity in BCa raised our attention. Therefore, we examined the cell type-based expression patterns of BCa-related genes [23] in female and male bladders. The results showed that BCa -related genes were predominantly expressed in urothelial cells in female and male bladders (Fig. 5A and B), suggesting that the urothelial cell is a cell type associated with BCa and maybe the earliest cell population to transform into tumor cells when BCa occurs, which is consistent with the results of previous reports. However, in our study, BCa-related genes expressed in the uroepithelium of women and men were inconsistent. The impact of this differential expression of BCa-associated genes requires further in-depth study.

## Discussion

Bladder is a vital component of the human urinary system and possesses important physiological functions. Some obvious sex differences in the incidence and prognosis of BCa and UTI suggest that there may be significant cellular heterogeneity in the female and male bladder, and uncover sex differences in the normal human bladder can help understand the epidemiological differences in UTI and BCa. However, studies on sex differences in the bladder were rarely reported, and the molecular mechanisms underlying these sex differences are not fully understood until now. In recent years, scRNA-seq has become a powerful tool to measure gene expression and explore cellular heterogeneity [24]. To explore the sex-based differences in bladder, we collected scRNA-seq data of six normal human bladders from public databases for further analysis. Our preliminary results indicated that sex-based differences in normal human bladders were predominantly located in urothelial cells, fibroblasts, B cells and T cells.

Urothelial cells are one of the most indispensable cellular barriers against uropathogens in the bladder. The apical face of urothelial cells is covered by uroplakin plaques; meanwhile, urothelial cells can secrete several antimicrobial compounds that prevent the invasion and adhesion of most uropathogens [25]. Consequently, sex disparities in urothelial cells may be an important factor influencing the difference in the incidence of UTIs between females and males. Therefore, we investigated the sex-based heterogeneity of urothelial cells in the bladders. The results showed that genes and pathways associated with ribosome biogenesis were mainly expressed and enriched in urothelial cells in male bladders compared with those in female bladders. Ribosome biogenesis is a significant driver of rapid cell growth [26]. The prevalence of UTIs is lower in men than in women. Anatomically, the female urethra is shorter and closer to the anus, causing the female bladder to be more susceptible to upstream infection from uropathogens. Our study may provide new insight into this difference at the cellular transcript level. The results suggested that the growth activity of male urothelial cells was higher than female urothelial cells. Active ribosome biogenesis in male urothelial cells can provide protein preparation for cell growth and proliferation, facilitating cellular self-renewal and repair, which are essential for constructing a complete cellular defense barrier. Therefore, the cellular barrier in the male bladder is more intact when facing uropathogens invasion, which may explain why the incidence of UTIs is lower in men than in women. However, ribosome biogenesis is also associated with tumorigenesis [27]. MDM2 can degrade the tumor suppressor p53 in cells via the ubiquitin-proteasome pathway [28]. When the rate of ribosome biogenesis increases, many ribosomal proteins bind rRNA to assemble ribosomes. At this time, the binding of ribosomal proteins to MDM2 is reduced, while the binding of MDM2 to the P53 protein is increased, leading to the degradation of P53 proteins in cells and increasing the risk of tumorigenesis, which may be one of the reasons for the higher incidence of BCa in men than in women.

The tumor microenvironment (TME), consisting of fibroblasts, extracellular matrix proteins, vascular vessels, and lymphocyte infiltration, is an important factor influencing tumor development and progression [29]. In tumor tissues, fibroblasts can be induced by tumor cells to transform into cancer-associated fibroblasts (CAFs), whose primary function is to maintain a TME conducive to tumor cell growth and proliferation and even foster malignancy [30, 31]. The collagen density of the TME often correlates with the invasiveness of tumor cells in several malignant tumors, such as breast cancer, pancreatic cancer, gastric cancer, and BCa [16, 32, 33]. In our study, we found that seven collagen genes associated with a poor prognosis of BCa were elevated in fibroblasts in the female bladder. When BCa occurs, these upregulated collagen gene may further mediate tumor cell infiltration into surrounding tissues and invasion into muscle tissue, resulting in a poor prognosis and increased mortality.

In general, females have a more robust immune response than males, resulting in different responses to many diseases between males and females. For example, females are more susceptible to autoimmune diseases, while males are more likely to suffer from fatal malignancies [18]. Similar results were observed in our study. In female bladder, we found more B-cell and T-cell activated signals and a higher expression of immunoglobulin genes. These results may represent a host change in females after frequent attacks by uropathogens. When an infection occurs, numerous immunoglobulins can assist the female bladder in recognizing uropathogens and clearing pathogens promptly. In contrast, such immune responses may be lacking in the male bladder, resulting in the delayed clearance of pathogens or even conversion to complicated UTI.

In addition, our work reveals commonalities and differences in the sex-based expression patterns of BCa-associated genes in normal human bladder cells. We found that BCa-related genes were predominantly expressed in urothelial cells in both males and females, suggesting that urothelial cells are a relevant cell population for BCa. However, there are also sex differences in the expression of BCa-related genes in urothelial cells, and further intensive studies are needed to reveal the impact of this differential expression.

Limitations of this study are the small number of samples and the lack of the analysis of scRNA-seq data from BCa and UTIs. Further research would benefit from high-quality scRNA-seq data from BCa and UTIs and enough histological evidence. Our study lays the foundation for understanding epidemiological differences in UTI and BCa.

## Conclusion

Taken together, our study describes a sex-based cellular landscape and gene expression differences in the normal human bladder at a single-cell resolution. We provide reasonable insight into sex disparities in the epidemiology of UTI and BCa, which will contribute to the understanding of epidemiological differences in UTI and BCa.

## Electronic supplementary material

Below is the link to the electronic supplementary material.

**Additional file 1. Supplementary Tables. Supplementary Table 1**. Samples information and sequencing statistics. **Supplementary Table 2**. Inclusion criteria of cells in different samples. **Supplementary Figure. 1**. Quality control (QC) of scRNA-seq data. (A-C) Violin plots illustrating the gene number (nFeature_RNA), percentage of mitochondrial genes (mt.pct), and percentage of hemoglobin genes (hb.pct) in each cell from different samples. **Supplementary Figure 2**. Analysis of cell activity for growth and proliferation in urothelial cells and pseudotime trajectory analysis of fibroblasts. (A-B) Cell cycle analysis of urothelial cells in females and males. The results show that 55.1% of male urothelial cells are in the S phase or G2/M phase, indicating that male urothelial cells may exist more proliferating cells. (C-D) TSNE plots and bar plot showing the proportion of PCNA-positive cells in the urothelial cells of females and males. More PCNA -positive cells exist in male urothelial cells. (E-F) Pseudotime trajectory analysis of female fibroblasts (E) and male fibroblasts (F).

## Data Availability

All data in this study were available in the public database. These data can be obtained from GEO database (GSE129845 and GSE134355), TCGA database (TCGA_BLCA), and CNGB database (CNS0094874 and CNS0094884).

## Abbreviations

UTI: Urinary tract infection
BCa: Bladder cancer
scRNA-seq: Single-cell RNA sequencing
ECM: Extracellular matrix
PCA: Principal component analysis
TSNE: T-distributed Stochastic Neighbor Embedding
DEGs: Differentially expressed genes
GO: Gene ontology
GSEA: Gene set enrichment analysis
SCENIC: Single-cell regulatory network inference and clustering
Fib: Fibroblast
Uro: Urothelial cell
Endo: Endothelial cell
SMC: Smooth muscle cell
Neutro: Neutrophil
Macro: Macrophage
TME: Tumor microenvironment
CAFs: Cancer-associated fibroblasts
TFs: Transcription regulators
