# Supplementary material for: Single-cell RNA sequencing reveals sexual diversity in the human bladder and its prospective impacts on bladder cancer and urinary tract infection
Source: BMC Med Genomics. 2023 Jun 5;16:122. doi: 10.1186/s12920-023-01535-6 (PMC10242993; doi:10.1186/s12920-023-01535-6)
Supplement: Supplementary file 1 — Additional file 1. Supplementary Tables. Supplementary Table 1. Samples information and sequencing statistics. Supplementary Table 2. Inclusion criteria of cells in different samples. Supplementary Figure. 1. Quality control (QC) of scRNA-seq data. (A-C) Violin plots illustrating the gene number (nFeature_RNA), percentage of mitochondrial genes (mt.pct), and percentage of hemoglobin genes (hb.pct) in each cell from different samples. Supplementary Figure 2. Analysis of cell activity for growth and proliferation in urothelial cells and pseudotime trajectory analysis of fibroblasts. (A-B) Cell cycle analysis of urothelial cells in females and males. The results show that 55.1% of male urothelial cells are in the S phase or G2/M phase, indicating that male urothelial cells may exist more proliferating cells. (C-D) TSNE plots and bar plot showing the proportion of PCNA-positive cells in the urothelial cells of females and males. More PCNA -positive cells exist in male urothelial cells. (E-F) Pseudotime trajectory analysis of female fibroblasts (E) and male fibroblasts (F). [file 12920_2023_1535_MOESM1_ESM.docx]

**Supplementary Tables**

**Supplementary Table 1. Samples information and sequencing statistics**

|  | **Gender** | **Age** | **Cell number** | **Data accession** |
| --- | --- | --- | --- | --- |
| B1 | male | 52 | 10000 | GSM3980126 |
| B2 | female | 32 | 10000 | GSM3980127 |
| B3 | female | 86 | 5015 | CNS0094874 |
| B4 | male | 56 | 6563 | CNS0094884 |
| B5 | female | 35 | 3560 | GSM3723358 |
| B6 | male | 47 | 9590 | GSM3723359 |

**Supplementary Table 2. Inclusion criteria of cells in different samples**

|  | **nFeature_RNA** | **mt.pct** | **hb.pct** |
| --- | --- | --- | --- |
| B1 | 200 < feature < 1000 | mt.pct < 25 | hb.pct < 1 |
| B2 | 200 < feature < 1000 | mt.pct < 25 | hb.pct < 1 |
| B3 | 500 < feature < 3000 | mt.pct < 25 | hb.pct < 1 |
| B4 | 500 < feature < 3000 | mt.pct < 25 | hb.pct < 1 |
| B5 | 1000 < feature < 3500 | mt.pct < 10 | hb.pct < 1 |
| B6 | 1000 < feature < 3500 | mt.pct < 10 | hb.pct < 1 |

**Figure Legends**

**Supplementary Figure. 1**. Quality control (QC) of scRNA-seq data. (A-C) Violin plots illustrating the gene number (nFeature_RNA), percentage of mitochondrial genes (mt.pct), and percentage of hemoglobin genes (hb.pct) in each cell from different samples.

**Supplementary Figure 2**. Analysis of cell activity for growth and proliferation in urothelial cells and pseudotime trajectory analysis of fibroblasts. (A-B) Cell cycle analysis of urothelial cells in females and males. The results show that 55.1% of male urothelial cells are in the S phase or G2/M phase, indicating that male urothelial cells may exist more proliferating cells. (C-D) TSNE plots and bar plot showing the proportion of *PCNA*-positive cells in the urothelial cells of females and males. More *PCNA* -positive cells exist in male urothelial cells. (E-F) Pseudotime trajectory analysis of female fibroblasts (E) and male fibroblasts (F).

**Supplementary Figures**

**Supplementary Figure 1**


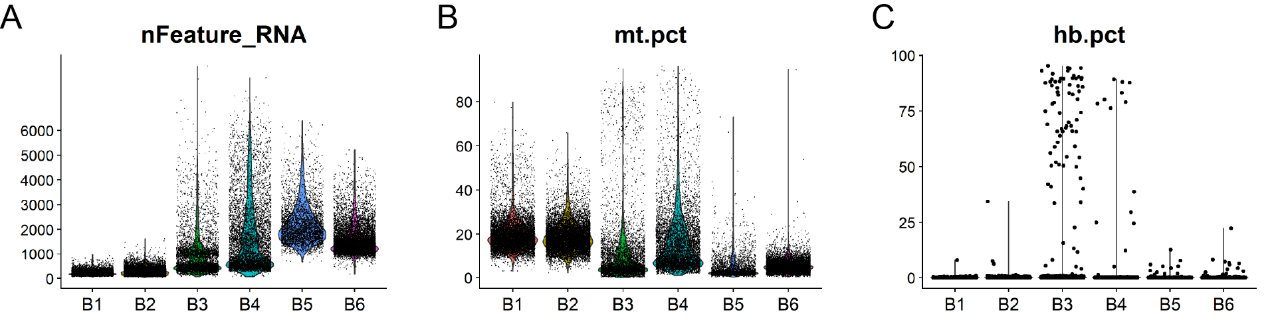


**Supplementary Figure 2**

**
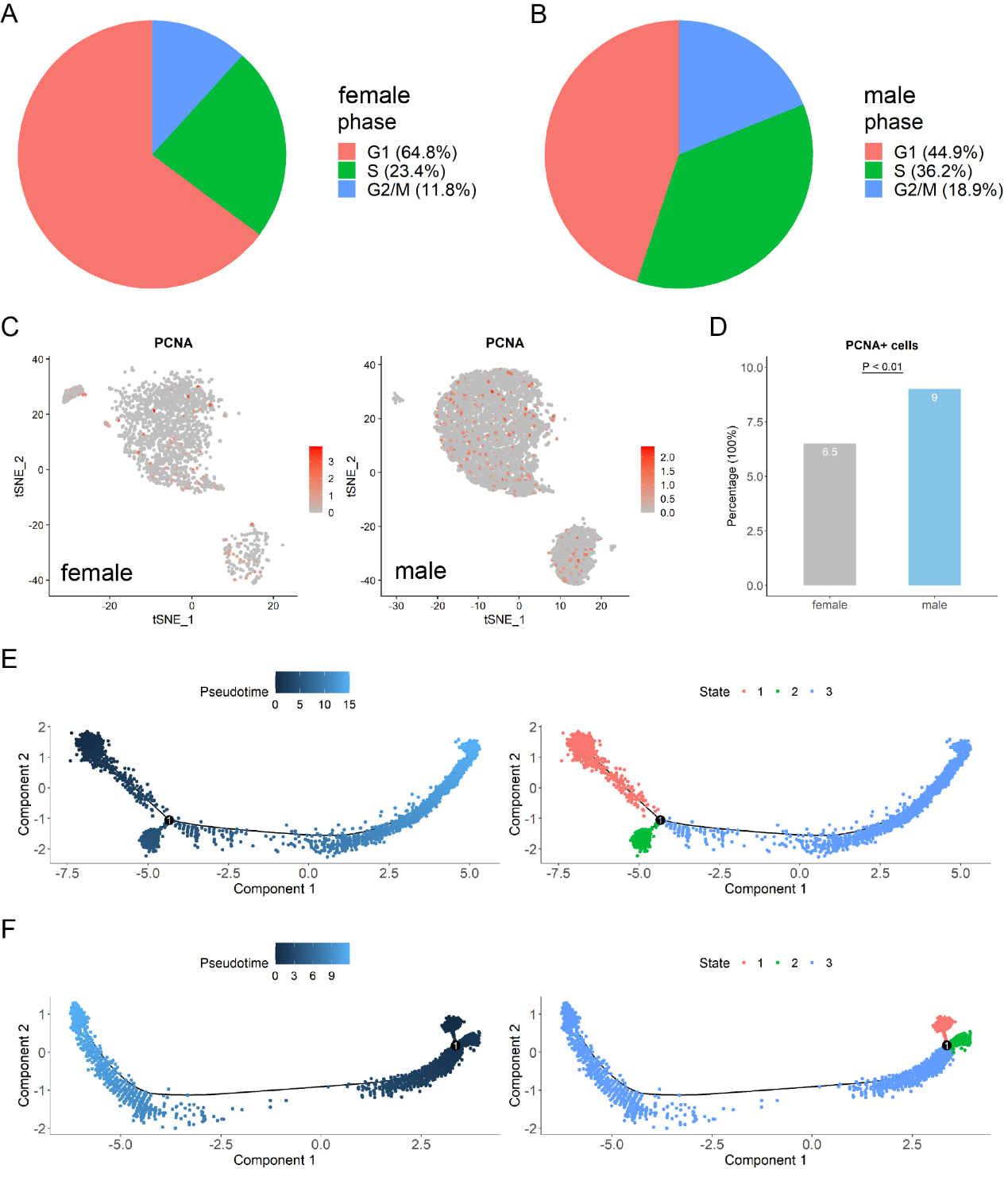
**
